# Aptamer Based SPREETA Sensor for the Detection of *Porphyromonas gingivalis* G-Protein

**DOI:** 10.4014/jmb.2310.10042

**Published:** 2023-12-11

**Authors:** Suk-Gyun Park, Hyun Ju Lee, Taeksoo Ji, Kyungbaek Kim, Seung-Ho Ohk

**Affiliations:** 1 Department of Oral Microbiology, School of Dentistry, Chonnam National University, Gwangju 61186, Republic of Korea; 2 Department of Cosmetic Science, Kwangju Women’s University, Gwangju 62396, Republic of Korea; 3 School of Electronics and Computer Engineering, Chonnam National University, Gwangju 61186, Republic of Korea

**Keywords:** Aptamer, SPREETA, biosensor, periodontal disease, *P. gingivalis*

## Abstract

We have developed an aptamer that specifically binds to *Porphyromonas gingivalis* to reduce the cellular damage caused by *P. gingivalis* infection and applied it as a biosensor. *P. gingivalis* is one of the major pathogens causing destructive periodontal disease among the periodontal microorganisms constituting complex biofilms. *Porphyromonas gingivalis* G-protein (PGP) known to play an important role in the transmission of germs was used as a target protein for the screening of aptamer. The aptamer that has binds to the G-protein of *P. gingivalis*, was screened and developed through the Systemic Evolution of Ligands by Exponential Energy (SELEX) method. Modified-Western blot analysis was performed with the aptamer which consisted of 38 single-stranded DNA to confirm the selectivity. ELONA (enzyme linked oligonucleotide assay) used to confirm that the aptamer was sensitive to PGP even at low concentration of 1 μg/ml. For the rapid detection of *P. gingivalis*, we constructed a surface plasmon resonance biosensor with SPREETA using the PGP aptamer. It was confirmed that PGP could be detected as low concentration as at 0.1 pM, which is the minimum concentration of aptamer sensor within 5 min. Based on these results, we have constructed a SPREETA biosensor based on aptamer that can bind to *P. gingivalis* G-protein. It can be used as an infection diagnosis system to rapidly diagnose and analyze oral diseases caused by *P. gingivalis*.

## Introduction

### Periodontal disease is a chronic bacterial infection that affects the gums and alveolar bone supporting the teeth [1]. Remaining food waste and the oral microbiome aggregate and stick to the surfaces of teeth, forming dental plaque. This plaque, combined with minerals, continues to grow around the gums and becomes dental calculus [2]. The plaque and calculus enlarge between the tooth and the gum, causing inflammation. This inflammation leads to gingivitis and periodontitis. In severe cases, it causes the alveolar bone to melt down, resulting in tooth loss

Particularly, anaerobic bacteria form biofilms in the mouth and contribute to periodontal disease [3, 4]. Gram negative anaerobic bacteria causing periodontal disease include *Porphyromonas gingivalis*, *Prevotella intermedia*, *Bacteroides forsythus*, *Treponema denticola* and *Campylobacter rectus* [5]. *P. gingivalis* is a major pathogen causing destructive periodontal disease among the microorganisms constituting complex biofilms [6]. *P. gingivalis* has been reported to be more toxic than other periodontal pathogenic strains, being involved in diseases such as chronic periodontitis through the production of proteases, collagenase, and cytotoxic metabolites [7].

The growth of these bacteria is closely related to the signaling pathway and is regulated by the extracellular environment and the growth stage of the cell itself. Extracellular signals, such as neurotransmitters and hormone growth factors, bind to their specific receptors, leading to phospholipid degradation of the cell membrane [8]. The degradation product of the phospholipid serves as a secondary transfer material, amplifying the signal transmitted from outside the cell and transmitting it to underlying signaling molecules. This signal transduction controls physiological phenomena such as cell growth, differentiation, and death.

G-protein-coupled receptors in bacterial signaling pathways are a group of receptors that carry a variety of signals, including hormones and neurotransmitters, that are involved in the physiological responses of various cells, from regulation of cAMP levels in cells to gene expression.

G-protein-coupled receptors in bacterial signaling pathways form a group of receptors that carry various signals, including hormones and neurotransmitters, involved in the physiological responses of various cells, from regulating cAMP levels to gene expression. The G-protein-coupled receptor signaling system operates when a hormone or neurotransmitter binds to a G-protein-coupled receptor, initiating the activation of other enzymes or ion channels in the cell via a special protein called G-protein (GTP-binding protein) [9, 10]. Bacterial G-proteins homologous to the essential *E. coli* Era protein have been identified in all bacterial genomes sequenced to date [11-14]. Era is known to be loosely associated with the inner membrane of *E. coli* [15].

In this study, an aptamer selectively binds to PGP(a signaling substance of *P. gingivalis*) was developed and utilized. Aptamers are single-stranded nucleic acids characterized by their ability to bind to a wide variety of target molecules with high affinity and specificity[16]. These aptamers have been applied in various fields, including therapeutic agents, diagnostic systems, drug delivery systems, sensors, and molecular imaging, with extensive research conducted [17-21]. If such an aptamer can be used as a diagnostic or therapeutic agent for *P. gingivalis*, it would make it easier to diagnose and treat periodontal disease and prevent other diseases caused by *P. gingivalis* infection.

We previously screened DNA aptamers specific to *Streptococcus mutans* and reported that these aptamers have the activity to block the adhesion of *S. mutans* to glass beads [22]. For the screening of aptamers specific to PGP, SELEX (Systemic Evolution of Ligands by EXponential Enrichment) method was employed in this study [23]. Among several aptamers screened by this method, one set of aptamers that showed specificity to PGP protein was selected and used to develop a biosensor system in this study.

In this research, we constructed a biosensor system using an aptamer and SPREETA, which is an electro-optical device utilizing surface plasmon resonance. SPREETA might provide real-time measurement of the binding of PGP to the aptamer attached to the sensor surface. An aptamer that selectively binds to PGP (*P. gingivalis* G-protein) was used as a bioreceptor and SPREETA was used as a signal processor. This aptamer-based biosensor system might provide a more effective and specific way for the real-time detection of *P. gingivalis* infection.

## Materials and Methods

### Bacterial Strains and Culture Conditions

The *P. gingivalis* ATCC 33277 was used as a control strain. The *pgp* gene was cloned and transformed into *E. coli* M15 (pQE-PGP) for the mass production of PGP protein [14]. Initially, *P. gingivalis* was inoculated in Brain Heart Infusion (BHI, Difco., USA) supplemented with 5 μg/ml of hemin and 0.5 μg/ml of vitamin K. The inoculum was then incubated at 37°C under anaerobic conditions (99.8% N_2_, 5% CO_2_, 10% H_2_, 85% N_2_ mixed gas) for 48 to 72 h. Subsequently, cells were collected by centrifugation at 16,000 ×*g* for 15 min at 4°C, washed three times with phosphate-buffered saline (PBS), and stored at -20°C.

### Aptamer

The aptamers utilized in this study were screened and developed through the SELEX method [23]. In brief, 5 μl of a PGP solution (10 μg/ml) was immobilized onto a PVDF membrane (3 × 3 mm), and the membrane was blocked with TBST buffer (0.2 M Tris-HCl pH 7.6, 1.37 M NaCl, 0.1% Tween 20) containing 2% skim milk (Seoul Milk Co., USA) for 30 min, followed by three washes. An aliquot of DNA library solution was boiled at 100°C for 5 min, immediately cooled on ice, and mixed with the PVDF membrane on which PGP was immobilized. After incubating for 1 h at 4°C on a rocking shaker, the membrane was washed three times with TTBS buffer and then subjected to subsequent PCR reaction. Following the amplification of aptamers bound to PGP, the nucleotide sequences of the aptamers were analyzed by Bioneer Co. Ltd. (Korea). The nucleotide sequences of the forward and reverse primers, as well as the DNA library, were listed in a previous report [22].

### Production of PGP

Recombinant PGP was prepared using the methods described in a previous report [14]. For the production of the PGP protein, *E. coli* M15 (pQE-PGP) was initially incubated overnight in 5 ml of LB (Luria Broth) medium supplemented with ampicillin and kanamycin. The cultured *E. coli* M15 (pQE-PGP) was then inoculated again in 50 ml of the same medium at 3% and incubated for 16 h in a shaking incubator. Subsequently, the cells were inoculated once more in 50 ml of the same medium and cultured for 2 h. After this 2-h incubation, 1 ml of the pellet was collected, and 1 mM Isopropyl β-D-1-thiogalactopyranoside (IPTG) was added to the remaining culture solution, followed by a 4-h incubation. The cultured *E. coli* M15 (pQE-PGP) was centrifuged at 15,000 ×*g* for 10 min to collect the cells, which were then used for purification.

### Protein Purification

The recombinant PGP protein was purified using the methods outlined in a previous report [14]. Ni-nitrilotriacetic acid (Ni-NTA; Qiagen Co., Germany) affinity column chromatography was employed for protein purification. Initially, 10 ml of lysis buffer (50 mM NaH_2_PO_4_, 300 mM NaCl, 10 mM imidazole, pH 8.0) was added to the cultured cells. The cells were disrupted with an ultrasonic grinder (Vibra cell VCX-600, Sonics and Materials Inc., USA) and centrifuged at 15,000 ×*g* at 4°C for 5 min.

The resulting supernatant was transferred to a new tube, and lysis buffer was added to achieve a final volume of 30 ml. Subsequently, 10 ml of Ni-NTA agarose was added, and the mixture was shaken at 4°C for 2 h to allow the protein to adsorb to Ni-NTA. The suspension was then filled into a column (14 × 5 mm) and carefully washed three times with 40 ml washing buffer (50 mM NaH_2_PO_4_, 300 mM NaCl, 20 mM imidazole, pH 8.0) and eluted 10 times with 5 ml of elution buffer (50 mM NaH_2_PO_4_, 300 mM NaCl, 250 mM imidazole, pH 8.0).

The protein concentration was quantified using the BCA Protein Assay Kit, and Bovine serum albumin (Sigma Co., USA) was employed as a standard sample. Absorbance was measured at 562 nm using a microplate reader (EZ Read 400, Biochrom, USA). The purity of the protein was confirmed by sodium dodecyl sulfate-polyacrylamide gel electrophoresis (SDS-PAGE; Bio-Rad Co., USA).

**Modified-Western blot analysis.** Western blot analysis [24] was conducted with a slight modification, using an aptamer instead of a primary antibody. The proteins from the SDS-PAGE gel were transferred to a polyvinylidene fluoride (PVDF) membrane in an electro-transfer buffer (25 mM Tris, 192 mM Glycine, 20% (v/v) methanol) for 90 min at 250 V, 350 mA. Subsequently, the membrane was blocked with TBST buffer (0.2 M Tris-HCl pH 7.6, 1.37 M NaCl, 0.1% Tween 20) containing 2% skim milk (Seoul Milk Co., USA) for 30 min and washed three times.

The washed PVDF membrane was then immersed in 20 ml of aptamer solution (used as a primary antibody) and allowed to react overnight at 4°C. After the reaction, the PVDF membrane was washed three times for 10 min in TBST buffer, and then it was reacted for 1 h in the secondary antibody solution (Anti-Digoxigenin-AP Fab fragments in TBST buffer) and washed three times for 10 min in TBST buffer. Finally, 200 μl of NBT/BCIP stock solution was added to 10 ml of detection buffer (0.1 M Tris-HCl, 0.1 M NaCl, pH 9.5) for the color reaction.

**Enzyme linked oligonucleotide assay (ELONA).** ELONA method [25] was employed with a slight modification for sensitivity analysis of the aptamer.The purified protein was diluted to the respective concentrations and dispensed into 96-well plates. These plates were allowed to stand at 4°C overnight to facilitate the attachment of proteins to the surface of the well plate.Subsequently, the remaining protein solution was removed, and the plates were blocked with TBST solution containing 2% skim milk for 30 min. After washing three times with TBST solution, diluted DIG-labeled aptamer was added at each concentration and allowed to react at 4°C for 1 h.

The plate was washed three times with TBST solution and then reacted with anti-DIG antibody diluted to 1:5,000 for 30 min. The color of the bound DIG was developed using NBT/BCIP as a substrate. Absorbance was measured at 562 nm using a microplate reader (EZ Read 400, Biochrom, USA).

**SPREETA system.** The SPREETA biosensor used in this study was a 3-channel SPREETA integrated with the ICx Nomadics SPR3 evaluation kit (Model 1146643) manufactured by Texas Instruments. The SPREETA biosensor comprises a 3-channel SPREETA sensor module, a 3-channel electronic controller (12 bit), an integrated flow block, a flow cell (1/16" OD, 6" long Teflon tubing), gasket, DB9 serial cable, DB15 serial cable (connecting the electronic controller to the integrated flow block), syringe pump, and a personal computer. The SPREETA biosensor system was established with several modifications [26]. The SPREETA biosensor system was assembled in a control box maintained at 30°C to ensure a constant temperature condition. It was connected to a personal computer, and multi-channel application software was operated with a fluid flow system.

The reference channel utilized distilled water, and based on this, the refractive index resulting from the binding of the ligand to the measurement liquid was calculated and converted.

### Detection of PGP with SPREETA Sensor

The detection of PGP with SPREETA biosensor system was followed by the methods of Marchesini et. al.[26] with a slight modification. The gold surface of the SPREETA sensor module was cleaned using a cleaning solution prepared by mixing Triton X-100 at a ratio of 1% to 0.1 N NaOH (w/v), followed by rinsing with distilled water to remove residual cleaning liquid. The refractive index (RI) reference value for distilled water on a cleaned gold surface is 1.33. The sensor module was installed and provided a hydrophilic environment suitable for biological samples. To prevent protein denaturation, a baseline buffer was injected into the sensing channel at a constant flow rate (0.1 ml/min) through a 1/8" diameter Teflon tube using a syringe pump for 5 min. The aptamer was then immobilized on the sensor, physically adsorbed to the gold surface by the interfacial hydrophilic effect.

After immobilizing the aptamer (a ligand substance) on the gold surface, PGP was injected at different concentrations (10 μg/ml, 5 μg/ml, and 1 μg/ml) for 10 min. Changes in Response Units (RU) due to the binding of PGP and ligand were measured using a personal computer and the Multiple Channel SPREETA program.

## Results

### Selection of Aptamer

Forty different aptamers binding to PGP were selected through the first round of SELEX, and each aptamer was subsequently amplified by PCR. These aptamers were then mixed at the same concentrations and utilized as a DNA library for another round of SELEX. This process was repeated to select an aptamer that could sensitively bind to PGP. After three rounds of SELEX screening, eight aptamers designated as AP-1 to AP-8 that bound to PGP were isolated. However, for reasons unknown (data not shown), the affinities of the aptamers to PGP were inconsistent. Among them, one aptamer that consistently exhibited affinity to PGP was selected for the development of an aptamer-based sensor. The nucleotide sequence of the aptamer used in this study is 5'-GGCGGCATAGCC CACTGTTGCCCCTGAAGTAATAAGGC-3'.

### Binding of Aptamer to PGP Protein

**Fig. 1:**
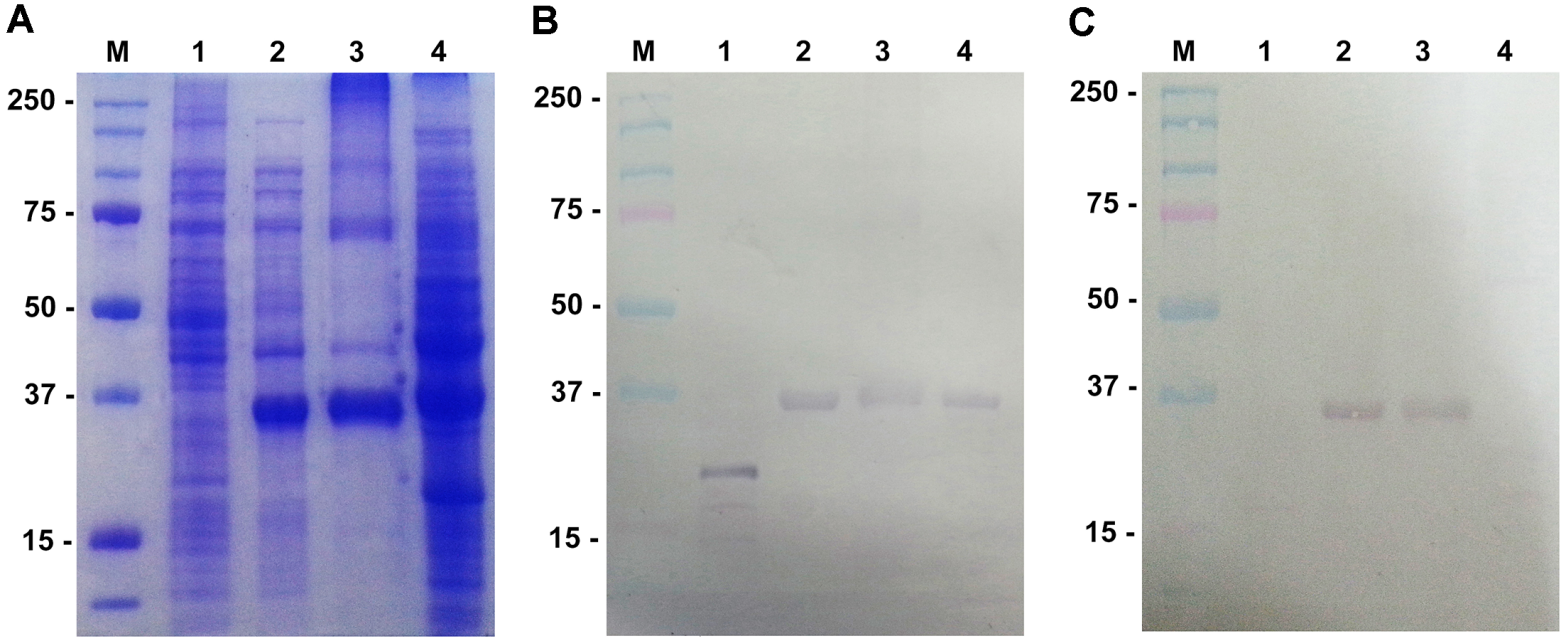
SDS-PAGE and modified-Western blot analysis of PGP specific aptamer. Bacterial extracts of *E. coli* M15 and *P. gingivalis* were applied to SDS-PAGE and their proteins were transferred to PVDF membranes for the Western blot analysis. Panels A, SDS-PAGE; B, modified Western blot analysis with 1 pM aptamer; C, with 0.1 pM aptamer. Lanes M, Molecular weigh marker (kDa); 1, the bacterial extract of *E. coli* M15; 2, the bacterial extract of *E. coli* M15 (pQE-PGP) induced with 1 mM IPTG; 3, partially purified PGP; 4, the bacterial extract of *P. gingivalis*.

To confirm the selectivity of the aptamer to the PGP protein, Modified-Western blot analysis was conducted (Fig. 1). The DIG-labeled aptamer demonstrated successive and selective binding to the PGP protein. At 1 pM of aptamer, clear binding was observed to the recombinant PGP (Fig. 1B), partially purified PGP from *P. gingivalis*, and the whole bacterial cell extract of *P. gingivalis*. However, non-specific binding to the bacterial extract of *E. coli* M15 at a different position (approximately 20 kDa) was evident. When 0.1 pM of aptamer was used, non-specific binding to the bacterial extract of *E. coli* M15 was not observed, as well as to the whole bacterial extract of *P. gingivalis* (Fig. 1C).

**Fig. 2:**
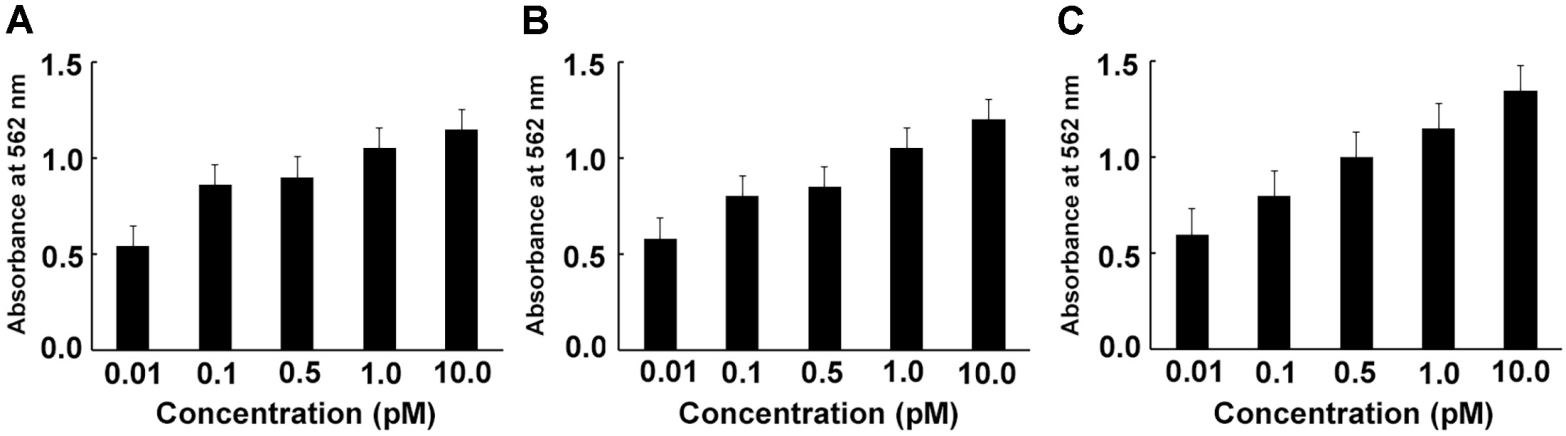
Enzyme linked oligonucleotide assay (ELONA) for the sensitivity assay of aptamer to PGP. The purified PGP protein was diluted to the respective concentrations and dispensed into 96-well plates. DIG-labeled aptamer was added at each concentration and reacted at 4°C for 1 h. Panels A, 1 μg/ml of PGP; B, 5 μg/ml of PGP; C, 10 μg/ml of PGP.

The binding aptamer to PGP was also monitored with for the range of 0.1 to 10 pM of aptamer (Fig. 2). ELONA is a modified version of the enzyme linked immunosorbent assay (ELISA), which uses aptamers instead of antibodies. The sensitivity of the aptamer was determined with three different concentrations of PGP such as 1 μg/ml, 5 μg/ml, and 10 μg/ml of PGP. The absorbance of the aptamer bound to the PGP was increased in a dose dependent manner from 0.01 pM to10 pM of aptamer, which reveals that aptamer has successfully bound to the PGP even in a low concentration as 0.01 pM. However, the absorbance of aptamer did not show any remarkable change with the increase of PGP concentration.

The binding of the aptamer to PGP was further monitored across the range of 0.1 to 10 pM of aptamer using ELONA (Fig. 2). ELONA is a modified version of the traditional ELISA, utilizing aptamers instead of antibodies. The sensitivity of the aptamer was evaluated at three different concentrations of PGP, namely 1 μg/ml, 5 μg/ml, and 10 μg/ml. The absorbance of the aptamer bound to the PGP increased in a dose-dependent manner from 0.01 pM to 10 pM of aptamer, demonstrating that the aptamer successfully bound to the PGP even at a low concentration of 0.01 pM. However, the absorbance of the aptamer did not exhibit any remarkable change with the increase in PGP concentration.

### Detection of PGP with Aptamer-Coated SPREETA Sensor

**Fig. 3:**
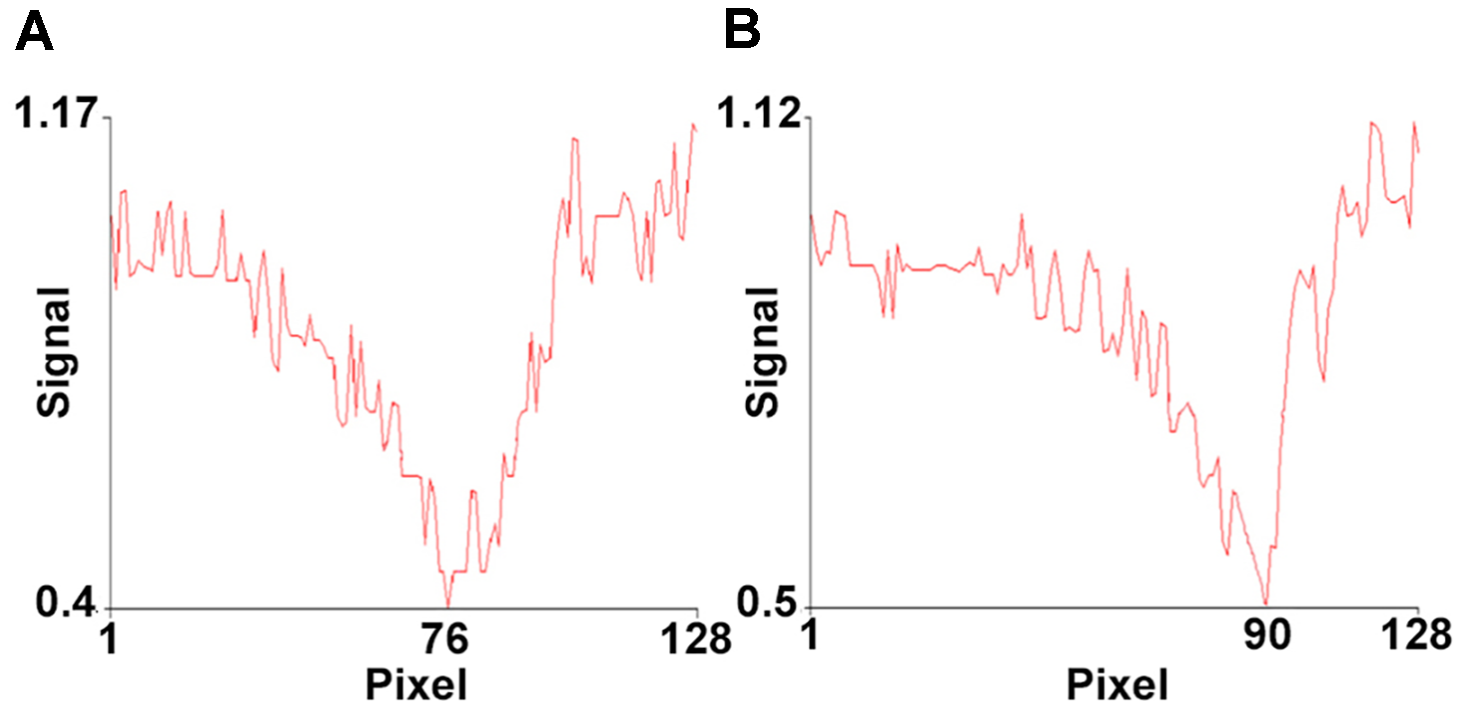
Verification of aptamer immobilization on the metal surfaces of SPREETA sensor. Immobilization of aptamer was carried out by the flow of aptamer solution at a flow rate of 0.1 ml/min through a 1/8" diameter Teflon tube using a syringe pump for 5 min. Panels A, SPREETA sensor with the immobilized aptamer; B, without aptamer.

To confirm the immobilization of the aptamer, a 1 μg/ml PGP solution was applied to the gold-coated metal surface of the SPREETA sensor. The pixel value of the SPR dip on the metal surface where the aptamer was immobilized was 76 (Fig. 3A), whereas that of the metal surface without the immobilized aptamer was 90 (Fig. 3B). As reported by Marchesin et. al. [26], the shift of SPR dip is attributed to the change in refractive index (RI) induced by the substances (aptamer in this report) on the chip surface. This confirmed that the aptamer was successfully immobilized on the metal surface of the SPREETA sensor.

**Fig. 4:**
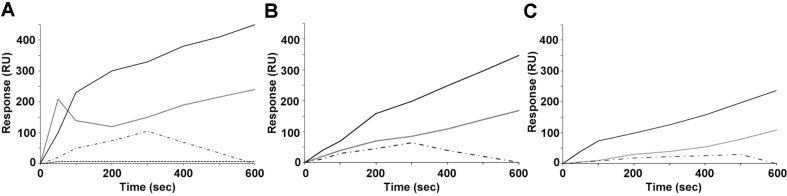
Responses of the SPREETA sensor with different concentrations of aptamer. After immobilization of aptamer on the metal surface of SPREETA sensor PGP was injected to flow with different concentrations for 10 min. Panels A, 10 μg/ml of PGP; B, 5 μg/ml of PGP; C, 1 μg/ml of PGP. Symbols ━, 10 pM of aptamer was immobilized on a metal surface; ━, 1 pM of aptamer; ■ · ■, 0.1 pM of aptamer; ■■■■, 0.01 pM of aptamer.

Subsequently, PGP solution was flowed on the metal surface of the SPREETA sensor, where the aptamer was immobilized, at various concentrations ranging from 0.01 pM to 10 pM. When a 10 μg/ml PGP solution was injected into the surface sensor with 10 pM of aptamer for 600 sec at a flow rate of 0.1 ml/min, the RU value rapidly increased until about 100 sec. Although the increasing rate slightly decreased after 100 sec, the RU value continued to gradually increase up to 450 RU until the end of the experiment (Fig. 4A). However, the increasing pattern of RU value with different concentrations of aptamer did not show the same trend as that of 10 pM. When 1 pM of aptamer was coated on the sensor surface, the RU value rapidly increased until 50 sec. After that, the RU value did not exhibit a proportional increase. In the case of 0.1 pM of aptamer, the RU value slightly increased until 300 sec and then gradually decreased. When 0.01 pM of aptamer was used in this sensor, it did not show any response to the flow of PGP solution.

With the flow of a PGP solution (5 μg/ml) through the sensor with 1 pM and 10 pM of aptamer, the RU value gradually increased until the end of the experiment. However, the final RU of the sensor showed a lower value than in the cases of 10 μg/ml of PGP (Fig. 4B). The same response pattern was observed in the case of a 1 μg/ml PGP solution, with even lower RU values of 240 and 110, respectively (Fig. 4C). In the case of 0.1 pM of aptamer used on the surface of the SPREETA sensor, it did not show any specific response pattern with either 10 μg/ml or 10 μg/ml of PGP.

## Discussion

*P. gingivalis* is known to cause destruction of periodontal tissues and bones by releasing virulence factors, including several proteases and lipopolysaccharide (LPS), leading to gingipains [27]. We previously reported the cloning of the *pgp* gene into *E. coli* M15 (pQE-PGP) and the purification of recombinant PGP protein [14]. In this study, an aptamer capable of selectively binding to PGP (the G-protein of *P. gingivalis*) was developed using the SELEX method. The binding efficiency of the aptamer to PGP was analyzed by Modified-Western blot analysis and ELONA assay method.

Modified-Western analysis used in this study with the aptamer provides a simple way of visualizing membranes compared to conventional Western blot analysis. Aptamers, consisting of a small chain of DNA or RNA nucleotides, are easy to label with color reagents such as digoxigenin, streptavidin, or other fluorescent reagents. Therefore, labeled aptamers might be used as substitutes for primary antibodies and directly visualized without a secondary antibody. In this report, we demonstrated that the binding of the aptamer to the PGP protein could be visualized in Western blot analysis without the need for a secondary antibody.

ELONA combines the principles of ELISA and nucleic acid hybridization to achieve sensitive and specific detection [25]. However, while the basic principles of ELONA remain consistent, specific protocols and modifications may vary depending on the target molecule and the desired application [28, 29]. The ELONA assay in this study showed that the higher the concentration of the aptamer, the better the binding capacity to PGP was observed. The sensitivity of the aptamer to PGP protein was confirmed at as low concentration as 0.01 pM. Both Modified-Western blot analysis and ELONA showed that the aptamer could specifically and sensitively bind to PGP. It is well reported that the concentration of the aptamer affects binding capacity [30].

On the other hand, the ultimate purpose of the biosensor is rapid and sensitive detection of target molecules. In this study, we constructed an aptamer-coated SPREETA sensor capable of quickly and accurately detecting *P. gingivalis*. Through the developed SPREETA sensor, we could detect PGP protein rapidly at a low concentration of 1 μg/ml with 0.1 pM of aptamer, although the lowest concentration of aptamer was 0.01 pM in Modified-Western blot analysis and ELONA. The difference in sensitivity between those methods might be due to the reaction time. The binding reaction between the aptamer and PGP was maintained overnight in Modified-Western blot analysis and ELONA, whereas it was completed within 600 sec in the SPREETA sensor system. It could be expected that the sensitivity of the sensor might be improved with further optimization of the immobilization efficiency of the aptamer.

The SPREETA system, is a biosensor technology that combines the principles of surface plasmon resonance (SPR) with electrochemical detection. It is designed for real-time, label-free monitoring of biomolecular interactions. When biomolecules (*e.g.*, aptamers, proteins) are immobilized on the gold-coated sensor surface, binding interactions with target molecules lead to changes in the SPR angle. These changes might be monitored by the changes in current or impedance associated with binding events.

This system is used for the rapid and sensitive detection of specific biomolecules, making it applicable in fields such as diagnostics, environmental monitoring, and bioanalytical research. One of the significant advantages is the label-free nature of detection, eliminating the need for fluorescent or radioactive labels. For the regeneration of the aptamer-coated sensor system, removing PGP bound to the aptamer-coated sensor system should be followed. However, during this process, a certain amount of aptamer that was coated on the gold surface might be removed as well, which might affect the consistency of the biosensor system. Therefore, it is important to develop a method for the rigid coating of the aptamer on the gold surface of the SPREETA sensor for the reliability of the sensor system.

In conclusion, we screened an aptamer that specifically binds to PGP and tested the binding ability using Modified-Western blot analysis and ELONA assay. Through these experiments, the selected aptamer showed efficient affinity and selectivity to PGP. Based on these results, we also constructed an aptamer-based SPREETA sensor system to detect PGP protein more rapidly and selectively. However, further optimization might be needed for the immobilization of the aptamer on the gold surface of the SPREETA sensor.
